# Inhibition of carrageenan-induced dental inflammatory responses owing to decreased TRPV1 activity by Dexmedetomidine

**DOI:** 10.1186/s12950-020-00245-5

**Published:** 2020-05-01

**Authors:** Gang Lv, Guanhua Zhu, Maohua Xu, Xingping Gao, Qingfeng Xiao

**Affiliations:** 1 Department of anesthesiology, Rizhao People’s Hospital, Rizhao, Shandong China; 2 Department of Anesthesiology, Jingzhou Central Hospital, Jingzhou, Hubei China; 3 Department of stomatology, Rizhao People’s Hospital, No. 126 Tai’an Road, Donggang District, Rizhao, 276800 China; 4 Department of Stomatology, Xiangyang Central Hospital, Affiliated Hospital of Hubei University of Arts and Science, No. 136, Jingzhou Street, Xiangyang, Hubei China

**Keywords:** Dental pulp cell, Inflammation, Dex, TRPV1, Cytokines

## Abstract

**Background:**

Dexmedetomidine (Dex) is a highly selective agonist of the α2 adrenergic receptor and a common sedative; however, its anti-inflammatory effect has been studied. In this study, the inhibitory effect of Dex on inflammation in dental pulp cells was assessed. For this, the effect of Dex on inflammation induced by carrageenan (Car) in human dental pulp cells (hDPCs) was evaluated. Car incubation induced a robust inflammatory response in hDPCs as well as activation of PKA–STAT3 and PKC–nuclear factor kappa B (NF-κB) signaling pathways.

**Results:**

Dex reduced the expression of inflammatory cytokines in a dose-dependent manner. Meanwhile, the phosphorylation of PKA, PKC, STAT3, and NF-κB as well as the nuclear accumulation of STAT3 and NF-κB were significantly increased in Dex-treated Car-induced hDPCs. Western blotting results also showed that the phosphorylation level of transient receptor potential cation channel subfamily V member 1 (TRPV1) was downregulated as a result of Dex treatment. Furthermore, we found that administration of the TRPV1 agonist capsaicin (Cap) reversed the effects of Dex on proinflammatory cytokines; however, the expression and activation of PKA–STAT3 and PKC–NF-κB signals were not altered owing to Cap administration.

**Conclusions:**

These results indicate that Dex plays a defensive role in dental pulp inflammation by regulating the TRPV1 channel and can be used as a potential target for human dental pulp inflammation intervention.

## Introduction

Pulp exposure and injury leads to pulpitis and induces severe inflammation, frequently resulting in persistent pain and referred pain. Dental pulp inflammation is a common phenomenon, usually a sequela of dental caries or trauma [1]. Clinically, it could cause severe pain [2], and if not controlled, it may eventually lead to fatal systemic inflammatory disease [3]. The mechanism of acute pulpitis is complex and involves repetitive trauma, inflammation, bacterial invasion, stimulation of the afferent nerve, secondary hyperalgesia, and in rare cases, periodontitis. Without effective treatment, the outcome is always root canal treatment. Therefore, considering the immediate effects of pulpitis, the identification of a new therapeutic target is significantly important for treating pulpitis. However, several studies have focused on the effect of immune cells [4], such as macrophages, dendritic cells, and lymphocytes.

Human dental pulp cells (hDPCs) are the main cell types present in dental pulp and play multiple roles in host defense and regeneration [5–7]. HDPCs induced by proinflammatory mediators, including tumor necrosis factor alpha (TNF-α) and lipopolysaccharide (LPS), can locally secrete numerous cytokines to attract additional immune cells and initiate and regulate inflammation [8, 9].

During inflammation, primary nociceptive neurons (nociceptors) are sensitized and the pain sensation (hyperalgesia) is increased. The direct effect of inflammatory mediators such as prostaglandins (PGI2 and PGE2) and sympathetic amines (epinephrine and dopamine) on their receptors in the nociceptor membrane can cause sensitization. Transient receptor potential cation channel subfamily V member 1 (TRPV1), a ligand-gated ion channel, is involved in pain modulation [10]. The flavonoid eriodictyol (an antagonist of the TRPV1 channel) also plays a part by reducing nociceptive behavior [11]. In addition, the nociceptor is partially characterized by the expression of TRPV1 [12].

As a specific agonist of the α2 adrenergic receptor, dexmedetomidine (Dex) is commonly used for analgesia and sedation purposes in the operation room and intensive care unit [13, 14]. Dex was recently reported to have a protective effect against inflammation that is triggered by endotoxin [15], spinal cord injury [16], sepsis [17], or lung injury [18]. In the present study, carrageenan (Car), an inflammation inducer [19], was used to induce pulp inflammation. STAT3 and NF-κB are common targets for IL-6-induced macrophages and carrageenan-induced mouse paw edema [20], while TRPV1 is crucial for pro-inflammatory STAT3 signaling [21]. Therefore, this study sought to study the protective effect of Dex on Car-induced pulp inflammation and determine the role of TRPV1, STAT3, and NF-κB on inflammation of hDPCs.

## Results

### Expression of proinflammatory cytokines induced by car in hDPCs

**Fig. 1:**
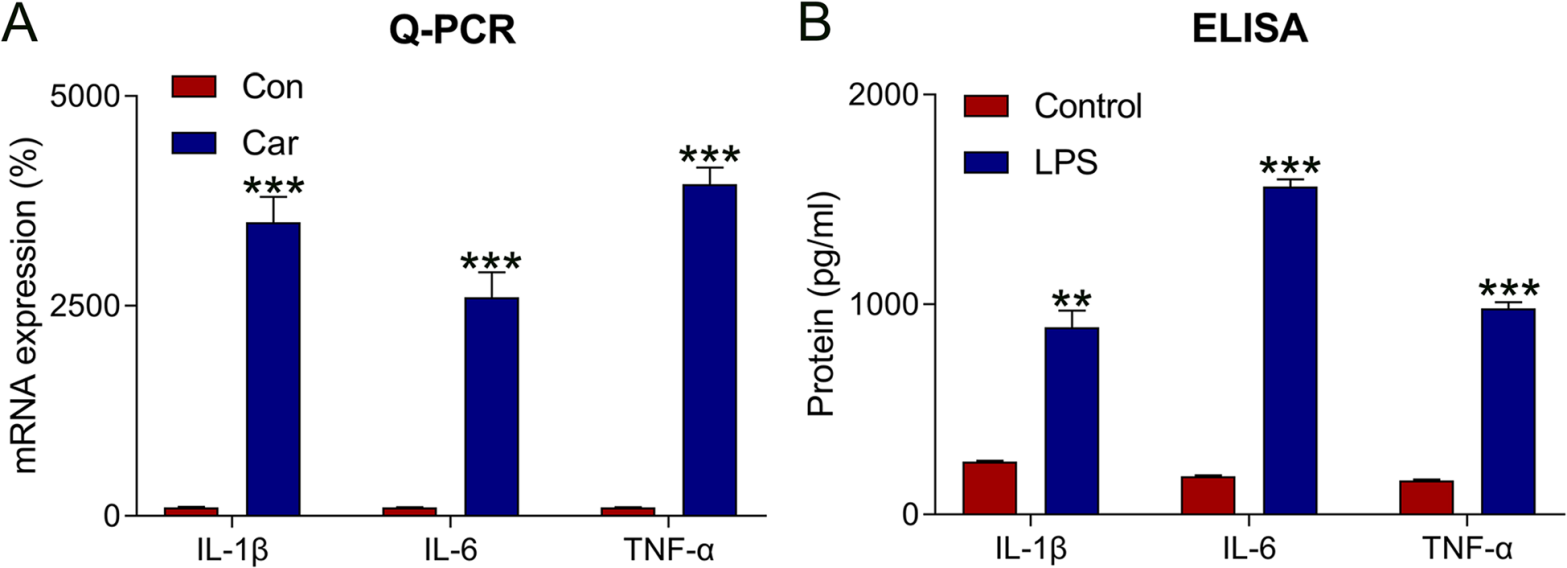
Car-triggered inflammation in hDPCs. hDPCs were treated with either 10 μM Car or PBS (as control) for 2 h. After lysis, the expressions of IL-1β, IL-6, and TNF-α were assessed by (**a**) qPCR and (**b**) ELISA. Data are expressed as means ± standard deviations. Comparisons between two groups were analyzed by *t*-test. **P < 0.01, ***P < 0.001

To explore hDPC inflammation following Car treatment, the expression of proinflammatory cytokines in hDPCs was assessed. qPCR and ELISA test results revealed that messenger RNA (mRNA) and protein expressions of IL-1β, IL-6, and TNF-α in HDPCs after Car treatment were higher than those in the control group (*P* < 0.01) (Fig. 1a and b).

### Car-induced activation of PKA–STAT3 and PKC–nuclear factor kappa B (NF-κB) in induced hDPCs

**Fig. 2:**
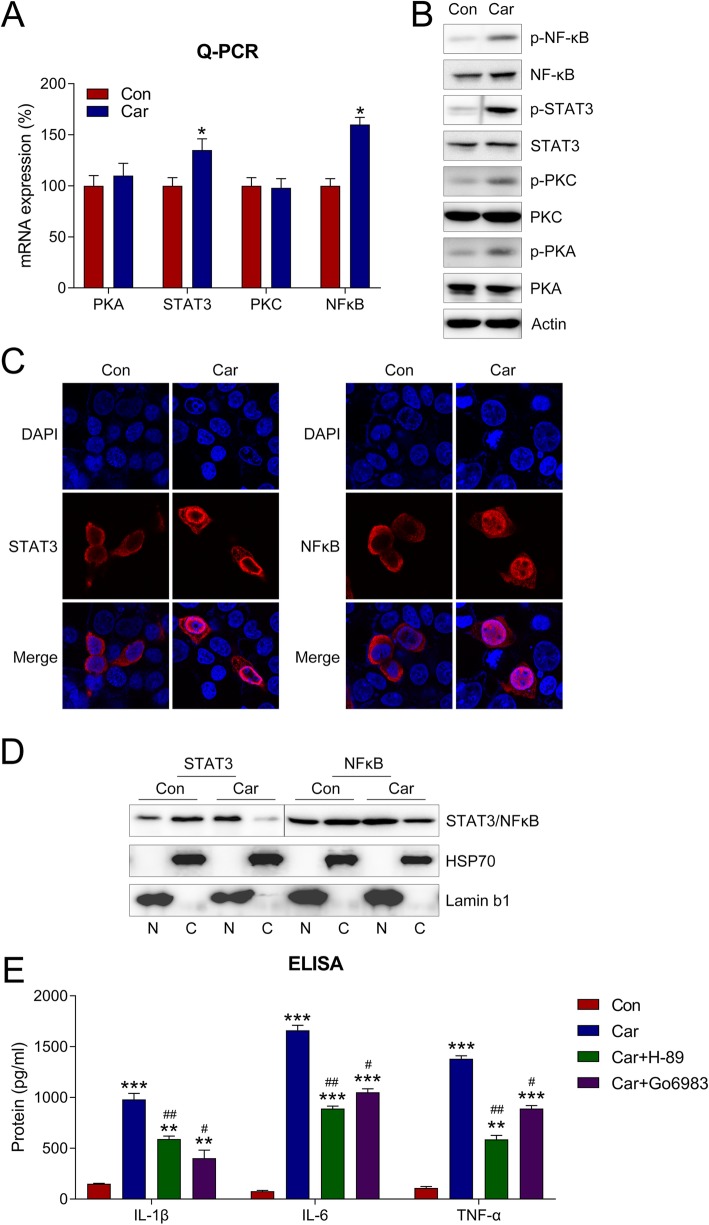
Car activated both PKA–STAT3 and PKC–NF-κB signaling in hDPCs. **a** Cell lysates were subjected to qPCR to assess the expressions of PKA, PKC, STAT3, and NF-κB. **b** WB was performed to probe with antibodies for phosphorylation and expression PKA, PKC, STAT3, and NF-κB in cell lysates. **c** IFA detected the cellular location of STAT3 and NF-κB in hDPCs. **d** Cell fractionation assay showed the location of STAT3 and NF-κB in nuclear and cytoplasmic fractions of hDPCs. **e** hDPCs were co-treated with either 100 μM Car and 10 μM H-89/Go6983 for 2 h. The expressions of IL-1β, IL-6, and TNF-α were assessed by ELISA. Data are expressed as means ± standard deviations. Comparisons between two groups were analyzed by *t*-test. *P < 0.05, **P < 0.01, ***P < 0.001 vs. Control group; ^#^*P* < 0.05, ^##^*P* < 0.01 vs. Car group

Because the activation of the PKA–STAT3 and PKC–NF-κB pathways is crucial for inducing cytokine expression [22, 23] the expression and phosphorylation of PKA, STAT3, PKC, and NF-κB after Car treatment were evaluated. qPCR results suggest that Car upregulates the mRNA expressions of STAT3 and NF-κB (*P* < 0.05), while the PKA and PKC expressions were not altered (Fig. 2a). Moreover, the WB results indicated that the levels of PKA, STAT3, PKC, and NF-κB phosphorylation were increased following Car treatment (Fig. 2B). Additionally, the nuclear localization of STAT3 and NF-κB was clearly increased as a result of Car incubation (Fig. 2c). Cellular fractionation was also performed to detect the nuclear (N) and cytoplasmic (C) distribution of STAT3 and NF-κB in hDPCs. The results showed that the “N” fraction of STAT3 and NF-kB in cells with Car treatment was increased, while the “C” fraction was reduced (Fig. 2d). These results suggest that Car treatment induced inflammatory reactions in hDPCs by activating the PKA–STAT3 and PKC–NF-κB pathways.

### Dex ameliorated inflammatory responses triggered by car

**Fig. 3:**
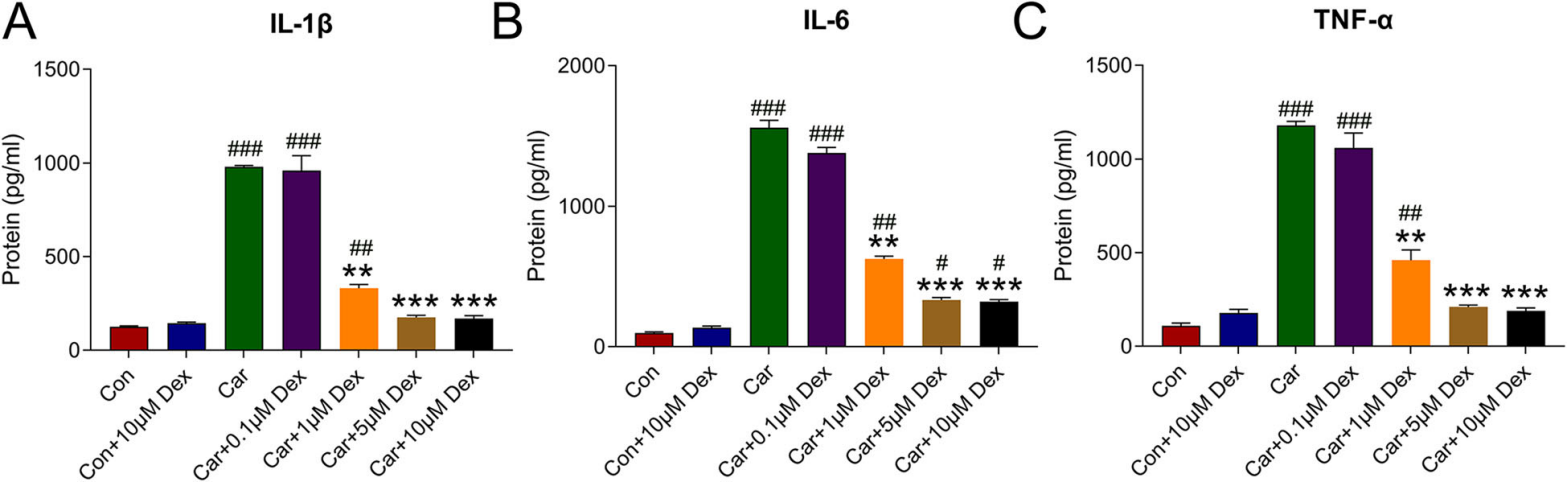
Dex ameliorated the generation of proinflammatory cytokines in hDPCs. hDPCs were co-treated with Car or different concentrations of Dex (0, 0.1, 1, 5, or 10 μM) for 2 h. The expressions of IL-1β (**a**), IL-6 (**b**), and TNF-α (**c**) at 2 h after Car and Dex treatment were detected by ELISA. Data are expressed as means ± standard deviations. Comparisons between multiple groups were analyzed by one way ANOVA. **P < 0.01, ***P < 0.001 vs. Car group; ^##^*P* < 0.01, ^***^P < 0.001 vs. Control group

Previous studies have shown that local Dex administration reverses inflammation induced by Car [24, 25]. Therefore, ELISA was utilized to detect the production of proinflammatory cytokines under Car stimulation and various concentrations of Dex. IL-1β, IL-6, and TNF-α were downregulated in a dose-dependent manner at 2 h after Dex treatment in hDPCs (*P* < 0.05) (Fig. 3a, b, and c).

**Fig. 4:**
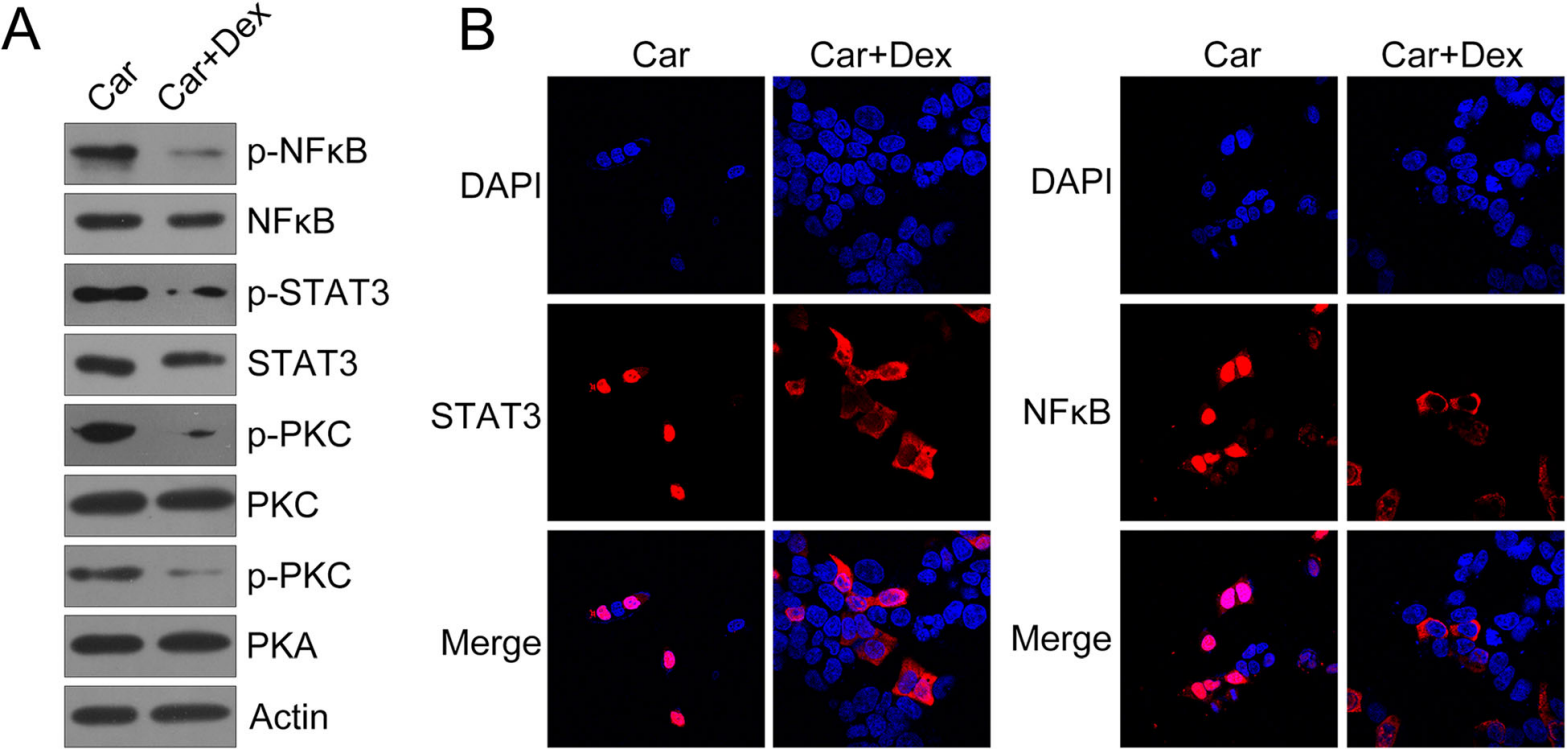
Dex deactivated both PKA–STAT3 and PKC–NF-κB signaling in hDPCs. hDPCs were cotreated with Car (100 μM) or Dex (5 μM) for 2 h. **a** WB was performed to probe with antibodies for the phosphorylation and expression of PKA, PKC, STAT3, and NF-κB in cell lysates. **b** IFA detected the cellular location of STAT3 and NF-κB in hDPCs

WB results showed that PKA, STAT3, PKC, and NF-κB phosphorylation was downregulated in hDPCs owing to stimulation using 5 μM Dex (*P* < 0.05) (Fig. 4a). Meanwhile, we found that STAT3 and NF-κB were redistributed in the cytoplasm following Dex treatment (Fig. 4b), suggesting that these two signaling pathways were blocked as a result of Dex treatment in hDPCs.

### Dex desensitized TRPV1 channel in car-treated hDPCs

**Fig. 5:**
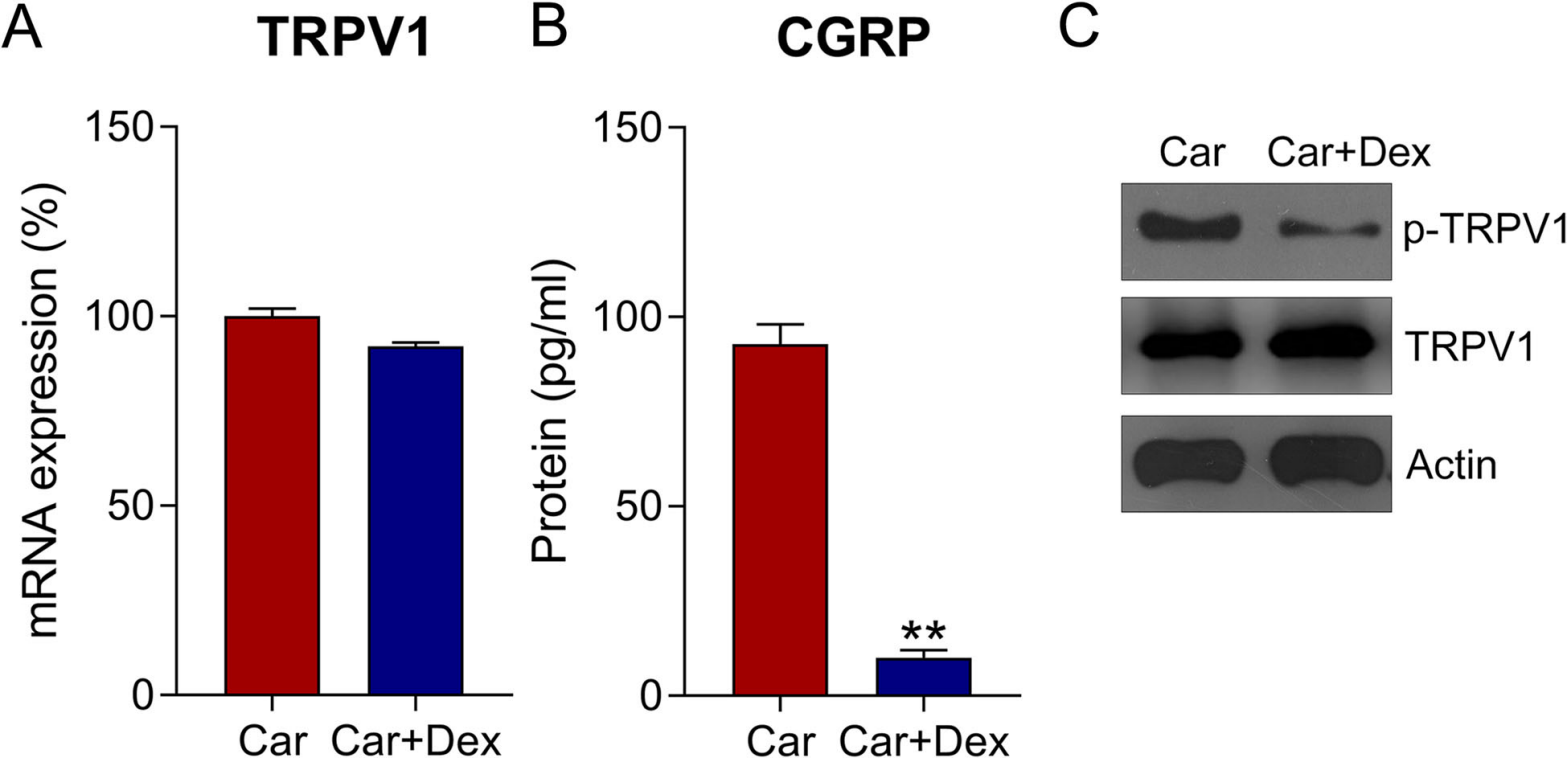
Dex desensitized the TRPV1 channel in hDPCs. **a** qPCR revealed the expression of TRPV1 in hDPCs. **b** The release of CGRP in cell culture medium was determined at 2 h after Car and/or Dex cotreatment by ELISA. **c** WB analyses determined the expression and phosphorylation of TRPV1 in hDPCs. Data are expressed as means ± standard deviations. Comparisons between two groups were analyzed by *t*-test. **P < 0.01

Previous studies have suggested that TRPV1 (a substrate of different protein kinases) is activated by multiple inflammatory mediators, such as PKC [26] and PKA [27]. Therefore, we attempted to examine the activation of TRPV1 during Car induction and Dex treatment. qPCR results showed that TRPV1 expression did not significantly change following Dex treatment (Fig. 5a); however, its activity, which was indicated by CGRP release, was clearly reduced after Dex treatment (Fig. 5b). Furthermore, WB indicated that TRPV1 phosphorylation was downregulated and confirmed that its expression did not change (Fig. 5c). The data suggested that TRPV1 was desensitized owing to Dex administration.

### Effects of the TRPV1 agonist cap on inflammation in hDPCs

**Fig. 6:**
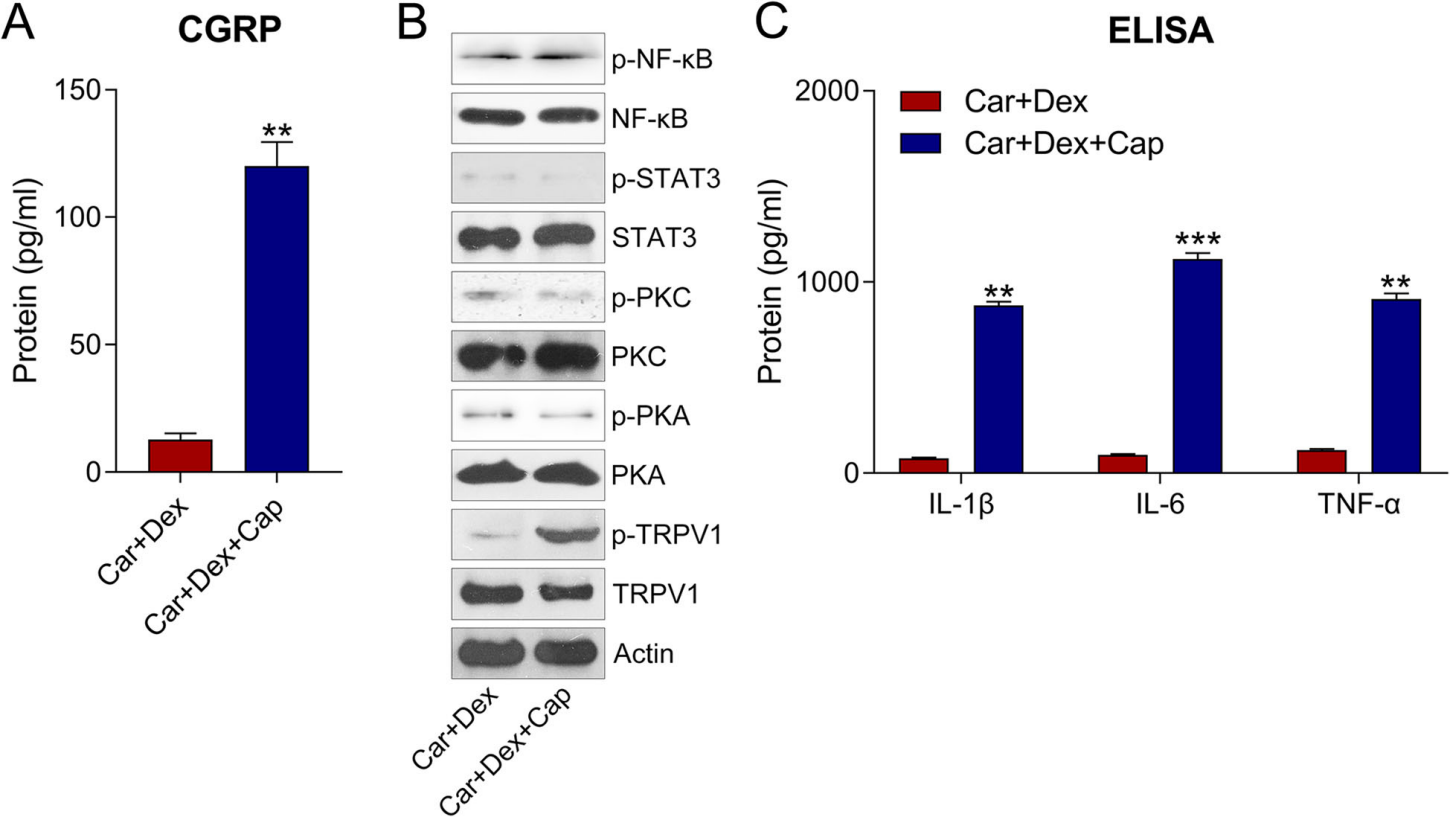
Cap treatment restored Car-induced inflammation in hDPCs. hDPCs were cotreated with Car (100 μM), Dex (5 μM), and/or Cap (5 μM) for 2 h. **a** The release of CGRP in cell culture medium was determined at 2 h after Car and/or Dex cotreatment by ELISA. **b** WB analyses were used to examine the phosphorylation and expression of PKA, PKC, STAT3, NF-κB, and TRPV1 in cell lysates. **c** ELISA was conducted to assess IL-1β, IL-6, and TNF-α expression. Data are expressed as means ± standard deviations. Comparisons between two groups were analyzed by *t*-test. **P < 0.01, ***P < 0.001

To further examine the influence of TRPV1 on Dex-ameliorated and Car-induced inflammation in hDPCs, cells were coadministered 5 μM of capsaicin (Cap), a TRPV1 agonist. We found that CGRP release was obviously increased after Cap treatment (*P* < 0.05) (Fig. 6a). In addition, TPRV1 phosphorylation was markedly increased after hDPCs were coadministered with Cap. Meanwhile, the activation of the PKA–STAT3 and PKC–NF-κB pathways was not altered, suggesting that TRPV1 was located downstream of these two pathways (Fig. 6b). In addition, the sensitization of TRPV1 resulted in the restoration of proinflammatory cytokine production (Fig. 6c), suggesting that TRPV1 sensitization counteracts the effects of Dex on hDPC inflammation.

## Discussion

Many molecules can be used to induce hDPC inflammation, including LPS, IL-6, IL-8, and TNF-α. More specifically, LPS can penetrate the dental pulp and induce inflammation and plays a key role in pulp infection [28]. Proinflammatory cytokines such as IL-6, IL-8, and TNF-α are usually detected in inflamed pulp and are considered key biomarkers and mediators for the diagnosis of pulp inflammation [29–31]. In the present study, Car was used to induce the inflammation of hDPCs. A previous study revealed that the Car-induced acute inflammatory response can be examined to elucidate the inflammatory period related to phagocyte infiltration, excessive production of free radicals, and release of inflammatory mediators, e.g., TNF-α, cyclooxygenase-2, and inducible nitric oxide synthase [32]. An in vivo model showed that Car-induced acute inflammatory response reached its peak at 2–3 h after injection, with the inflammation later disappearing within 24–74 h [33]. The inflammatory response induced by Car is considered to be a biphasic situation in which multiple mediators induce the generation of inflammation [34]. In this study, the robust generation of proinflammatory cytokines, i.e., IL-1β, IL-6, and TNF-α, and the phosphorylation and nuclear localization of STAT3 and NF-κB were observed after 100-μM Car treatment for 2 h. These findings indicated the successful induction of dental pulp inflammation by Car.

Existing evidence has revealed that TRPV1 is involved in the occurrence and development of immune-related diseases and is a therapeutic target that is easily blocked by small molecules. For instance, TRPV1 is currently considered to be an anticipated molecular target for the clinical evaluation of epilepsy therapy [35]. Its antagonists are employed for the treatment of pruritus, inflammation-related pain, tissue damage, ischemia, and other diseases [36, 37], and its agonists have been used in a phase III clinical trial for the treatment of cluster headache, analgesia, and osteoarthritis [38, 39]. Various inflammatory mediators can sensitize TRPV1, including neurotransmitters, cytokines and chemokines, lipids, peptides or small proteins, and growth factors. Many inflammatory mediators enhance the activity of TRPV1 [40]. TRPV1 is lowly expressed in pulpal sensory neurons relative to their expression in the pulpal trigeminal ganglia [41]. LPS treatment in dental trigeminal ganglia induced pulpitis by upregulating expression of the TRPV1 channel [42]. Previous studies have shown that local Dex administration reverses inflammation induced by Car [22, 23]. In the present study, treatment using Dex led to impaired inflammation and deactivation of the TRPV1 channel. Further usage of the TRPV1 agonist Cap restored the generation of proinflammatory cytokines, suggesting that the TRPV1 channel was involved in the Car-triggered inflammation of DPCs.

Previous studies demonstrated that TRPV1 can be activated by inflammatory mediators and corresponding receptors, i.e., PKC [26] and PKA [27]. Both kinases can phosphorylate TRPV1 at different serine and threonine residues, resulting in TRPV1 sensitization. Alternatively, the calcium signaling pathway can be activated and TRPV1 phosphorylation can be induced by increased intracellular calcium in the TRPV1 channel, leading to downstream activation of STAT3 [21] and NF-κB and cytokine production [43]. STAT3, a member of the STAT protein family, is an important regulator in tumor cells and plays a critical role in inflammation and tumorigenesis by regulating cell metabolism [44]. STAT3 protein exists in an inactive form in the cytoplasm and can be activated by associated kinases and phosphorylated at multiple phosphorylation sites [45]. The most common form of NF-κB is a heterodimer of p50 and p65/RelA proteins. Similarly, in the deactivated condition, NF-κB is present in an inactive form that is retained in the cytoplasm by the inhibitory protein IκB. Both STAT3 and NF-κB are transcription factors that are involved in immune responses during inflammation [22, 23]. Previous studies have demonstrated that STAT3 [46, 47] and NF-κB [22, 48–51] can be upregulated and activated in the inflammatory DPCs or dental pulp stem cells. In the present study, PKA–STAT3 and PKC–NF-κB signaling was activated by Car administration, while Dex treatment clearly reduced the phosphorylation and nuclear subcellular location of STAT3 and NF-κB, indicating that STAT3 and NF-κB activation might be attributed to Car-induced inflammation of DPCs. However, both signals were not altered after Cap administration, indicating that TRPV1 activation occurs downstream of these two signals.

## Conclusions

In summary, our study demonstrated the intrinsic mechanism underlying dental pulp inflammation in hDPCs. Car can induce inflammation by sensitization of TRPV1 via the PKA–STAT3 and PKC–NF-κB pathways, which can be ameliorated by Dex. The present study shows that Dex can be used as a potential drug for gingivitis, which can induce anti-inflammatory potency in dental pulp cells.

## Methods

### Ethics

All experiments were approved by the Ethics Committee of Rizhao People’s Hospital. All subjects signed informed consent forms.

### Separation and culture of hDPCs

Healthy permanent premolars for orthodontic or impacted third molars were collected from subjects aged 18–26 years. As mentioned above, hDPCs were separated and cultured using a previously described enzymatic method [52]. The dental pulp tissue was separated, cut into small pieces, and digested at 37 °C for 20 min with 3 mg/mL of type I collagenase (Gibco, USA). Next, the chopped pulp tissue was cultured at 37 °C in Dulbecco’s modified Eagle’s medium (DMEM) containing 20% fetal bovine serum, 100 U/mL of penicillin (Gibco, USA), and 100 mg/mL of streptomycin with 5% CO_2_. The medium was replaced every 3 days. After the cells achieved 80% confluence, they were separated by trypsin/ethylenediaminetetraacetic acid (Gibco, USA) and sub-cultured at a ratio of 1:2.

### Dex incubation

Dex (1,179,333, Sigma, 350 μM) was frozen at − 20 °C and diluted with DMEM/F-12 to a specified concentration if necessary. Before Dex treatment, cells were allowed to reach approximately 70–80% confluence. The cells were exposed to Dex at various concentrations (0, 0.1, 1, 5, or 10 μM) for 2 h to determine the optimal concentration. Cells were then divided into the following groups: control group, in which the cells were incubated without Dex treatment in a humidified environment at 37 °C with 5% CO_2_; Car group, in which the cells were incubated with 100 μM of Car for 2 h; and Dex/Car group, in which the cells were pretreated with 5 μM of Dex and 100 μM of Car for 2 h.

### Drug administration

To induce an inflammatory response, cells were incubated with Car (C1013, Sigma, 100 μM) for 2 h. To promote TRPV1 activity, cells were incubated with capsaicin (Cap, 21,750, Sigma, 5 μM) for 2 h. To inhibit PKA activity, cells were treated with H-89 (B1427, Sigma, 10 μM) for 2 h. To inhibitor PKC activity, cells were incubated with Go6983 (ab144414, Abcam, 10 μM) for 2 h.

### Immunofluorescence assay (IFA)

hDPCs were inoculated in a 24-well plate and fixed with 4% polyformaldehyde (28,906, Thermofisher) for 15 min. hDPCs were permeated for 30 min with 0.1% Triton X-100, cultured at ambient temperature for 15 min with 10% goat serum, and treated at 4 °C with the primary antibody overnight. hDPCs were washed with PBS three times and incubated at ambient temperature for 1 h with the secondary Cy3-labeled antibody in dark conditions. Then, the cells were stained with 4′,6-diamidino-2-phenylindole (D1306, Thermofisher) for 15 min. Images were obtained at 400× magnification using a fluorescent microscope.

### Subcellular fractionation

Cells (1 × 10^6^) were plated on 12-cm dishes and grown for 36 h. Then, the cells were harvested via scraping into 500 μL of cell lysis buffer containing 10 mM HEPES (pH 7.4), 10 mM NaCl, 1 mM KH_2_PO_4_, 5 mM NaHCO_3_, 1 mM CaCl_2_, 0.5 mM MgCl_2_, and 5 mM EDTA with complete protease inhibitor cocktail. Cells were allowed to swell for 5 min, followed by Dounce homogenization for 50-time strokes. The cells were then centrifuged at 5000 rpm for 5 min, generating a pellet containing nuclei and debris and a supernatant of cytosol and plasma. Pellets were resuspended in 1 mL of buffer containing 10 mM Tris (pH 7.5), 300 mM sucrose, 1 mM EDTA, and 0.1% NP40 with complete protease inhibitor cocktail and then pelleted, resuspended, and washed twice. The final pellets obtained were pure nuclei.

### Western blotting (WB)

The cell lysis buffer was used for the lysis of hDPCs. Protein was determined using a bicinchoninic analysis kit, separated by 10% sodium dodecyl sulfate–polyacrylamide gel electrophoresis, and then transferred to a polyvinylidene fluoride or polyvinylidene difluoride membrane. Tween 20 was added to bovine serum albumin (BSA; 5%) phosphate buffer to block nonbinding sites on the membrane for 1 h. Protein was cultured at 4 °C overnight with the primary antibody (p65, ab16502, Abcam, 1:1000; STAT3, ab5073, Abcam, 1:1000; PKC, ab19031, Abcam, 1:2500; PKA, ab187515, Abcam, 1:5000; TRPV1, PA1–748, Thermofisher, 1:1000; Phospho NF-kB p65 (S536), ab86299, Abcam, 1:500; Phospho STAT3 (S727), ab30647, Abcam, 1:500; Phospho PKC (T497), ab59411, Abcam, 1:1000; Phospho PKA alpha (Ser338), PA5–64489, Thermofisher, 1:500; Phospho TRPV1 (Ser503), PA5–64860, Thermofisher, 1:200; Actin, ab8227, Abcam, 1:5000; HSP70, ab2787, Abcam, 1:1000; Lamin B1, ab65986, Abcam, 1:1000), and the secondary antibody was bound to HRP (ab205718, ab205719, Abcam). The protein bands were stained, and the gray values were measured on a C-DiGit Blot Scanner.

### RNA extraction and quantitative real-time polymerase chain reaction (qPCR)

Total RNA was extracted with glyceraldehyde 3-phosphate dehydrogenase (GAPDH) used as an internal standard. Next, under the following conditions, RNA was detected using qPCR (using an SYBR-Green Kit) in a 20-μL system: predenaturation (95 °C, 10 min), denaturation (95 °C, 15 s, 40 cycles), annealing (60 °C, 30 s), and extension (72 °C, 30 s). Quantitative analysis was based on the 2^−ΔΔCT^ method and normalized according to GAPDH. The sequences of primers used in this study was displayed as follows: IL-1β F: 5′-CCA CAG ACC TTC CAG GAG AAT G-3′, IL-1β R: 5′-GTG CAG TTC AGT GAT CGT ACA GG-3′; IL-6 F: 5′-AGA CAG CCA CTC ACC TCT TCA G-3′, IL-6 R: 5′-TTC TGC CAG TGC CTC TTT GCT G-3′; TNF-α F: 5′-CTC TTC TGC CTG CTG CAC TTT G-3′; TNF-α R: 5′-ATG GGC TAC AGG CTT GTC ACT C-3′; PKA F: 5′-CAT ATT GCC GAA CAG ATT GG-3′, PKA R: 5′-GCT GGA CTT CAT TGG CTG TA-3′; PKC F: 5′-CGA CTG TCT GTA GAA ATC TGG-3′, PKC R: 5′-CAC CAT GGT GCA CTC CAC GTC-3′; STAT3 F: 5′-CTT TGA GAC CGA GGT GTA TCA CC-3′, STAT3 R: 5′-GGT CAG CAT GTT GTA CCA CAG G-3′; NF-κB F: 5′-GCA GCA CTA CTT CTT GA-3′, NF-κB R: 5′-TCT GCT CCT GAG CAT TG-3′; TRPV1 F: 5′-CCA CAG CGG TGG TGA CGC-3′, TRPV1 R: 5′-GGA GCT GTC AGG TGG CCG-3′; GAPDH F: 5′-GCA CCG TCA AGG CTG AGA A-3′, GAPDH R: 5′-TGG TGA AGA CGC CAG TGG A-3′.

### Enzyme-linked immunosorbent assay (ELISA)

According to the manufacturer’s instructions, the concentrations of interleukin (IL)-1β (BMS224–2, Thermofisher), IL-6 (EH2IL6, Thermofisher), TNF-α (KHC3011, Thermofisher), and CGRP (ABIN1095216, Antibodies-online) in the cell culture supernatants were analyzed using an ELISA kit. An automated microplate reader (SpectraMax® M5) was used for the measurement of the optical density (OD) at 450 nm. The concentrations of each sample were detected based on optical density and the concentration of the standard.

### Statistical analysis

The results of our study are presented as means ± standard deviations. Comparisons between two groups or multiple groups were analyzed using one-way ANOVA or a two-tailed Student’s *t*-test, respectively. A *P*-value of < 0.05 was considered to indicate a statistically significant difference.

## Data Availability

The datasets used and/or analyzed during the current study are available from the corresponding author on reasonable request.

## References

[1] Boyle M, Chun C, Strojny C, Narayanan R, Bartholomew A, Sundivakkam P (2014). Chronic inflammation and angiogenic signaling axis impairs differentiation of dental-pulp stem cells. PLoS One.

[2] Lin J-J, Du Y, Cai W-K, Kuang R, Chang T, Zhang Z (2015). Toll-like receptor 4 signaling in neurons of trigeminal ganglion contributes to nociception induced by acute pulpitis in rats. Sci Rep.

[3] Serhan CN, Petasis NA (2011). Resolvins and protectins in inflammation resolution. Chem Rev.

[4] Renard E, Gaudin A, Bienvenu G, Amiaud J, Farges J, Cuturi M (2016). Immune cells and molecular networks in experimentally induced pulpitis. J Dent Res.

[5] Lee S-I, Min K-S, Bae W-J, Lee Y-M, Lee S-Y, Lee E-S (2011). Role of SIRT1 in heat stress-and lipopolysaccharide-induced immune and defense gene expression in human dental pulp cells. J Endod.

[6] Xiong H, Wei L, Peng B (2015). IL-17 stimulates the production of the inflammatory chemokines IL-6 and IL-8 in human dental pulp fibroblasts. Int Endod J.

[7] Zhao Y, Wang C-L, Li R-M, Hui T-Q, Su Y-Y, Yuan Q (2014). Wnt5a promotes inflammatory responses via nuclear factor κB (NF-κB) and mitogen-activated protein kinase (MAPK) pathways in human dental pulp cells. J Biol Chem.

[8] Feng Z, Li Q, Meng R, Yi B, Xu Q (2018). METTL 3 regulates alternative splicing of MyD88 upon the lipopolysaccharide-induced inflammatory response in human dental pulp cells. J Cell Mol Med.

[9] Horst OV, Horst JA, Samudrala R, Dale BA (2011). Caries induced cytokine network in the odontoblast layer of human teeth. BMC Immunol.

[10] Basbaum AI, Bautista DM, Scherrer G, Julius D (2009). Cellular and molecular mechanisms of pain. Cell..

[11] Rossato MF, Trevisan G, Walker CIB, Klafke JZ, de Oliveira AP, Villarinho JG (2011). Eriodictyol: a flavonoid antagonist of the TRPV1 receptor with antioxidant activity. Biochem Pharmacol.

[12] Caterina MJ, Schumacher MA, Tominaga M, Rosen TA, Levine JD, Julius D (1997). The capsaicin receptor: a heat-activated ion channel in the pain pathway. Nature.

[13] Cui J, Zhao H, Yi B, Zeng J, Lu K, Ma D (2015). Dexmedetomidine attenuates bilirubin-induced lung alveolar epithelial cell death in vitro and in vivo. Crit Care Med.

[14] Gertler R, Brown HC, Mitchell DH, Silvius EN (2001). Dexmedetomidine: a novel sedative-analgesic agent. Baylor university medical center proceedings. Taylor Francis.

[15] Taniguchi T, Kidani Y, Kanakura H, Takemoto Y, Yamamoto K (2004). Effects of dexmedetomidine on mortality rate and inflammatory responses to endotoxin-induced shock in rats. Crit Care Med.

[16] Can M, Gul S, Bektas S, Hanci V, Acikgoz S (2009). Effects of dexmedetomidine or methylprednisolone on inflammatory responses in spinal cord injury. Acta Anaesthesiol Scand.

[17] Tasdogan M, Memis D, Sut N, Yuksel M (2009). Results of a pilot study on the effects of propofol and dexmedetomidine on inflammatory responses and intraabdominal pressure in severe sepsis. J Clin Anesth.

[18] Yang C-L, Tsai P-S, Huang C-J (2008). Effects of dexmedetomidine on regulating pulmonary inflammation in a rat model of ventilator-induced lung injury. Acta Anaesthesiol Taiwanica.

[19] Nantel F, Denis D, Gordon R, Northey A, Cirino M, Metters KM (1999). Distribution and regulation of cyclooxygenase-2 in carrageenan-induced inflammation. Br J Pharmacol.

[20] Basu A, Das AS, Sharma M, Pathak MP, Chattopadhyay P, Biswas K (2017). STAT3 and NF-κB are common targets for kaempferol-mediated attenuation of COX-2 expression in IL-6-induced macrophages and carrageenan-induced mouse paw edema. Biochemistry Biophys Rep.

[21] Yoshida A, Furube E, Mannari T, Takayama Y, Kittaka H, Tominaga M (2016). TRPV1 is crucial for proinflammatory STAT3 signaling and thermoregulation-associated pathways in the brain during inflammation. Sci Rep.

[22] Chang J, Zhang C, Tani-Ishii N, Shi S, Wang C-Y (2005). NF-κB activation in human dental pulp stem cells by TNF and LPS. J Dent Res.

[23] Takeda K, Akira S (2000). STAT family of transcription factors in cytokine-mediated biological responses. Cytokine Growth Factor Rev.

[24] Sukegawa S, Higuchi H, Inoue M, Nagatsuka H, Maeda S, Miyawaki T (2014). Locally injected dexmedetomidine inhibits carrageenin-induced inflammatory responses in the injected region. Anesth Analg.

[25] Walker SM, Howard RF, Keay KA, Fitzgerald M (2005). Developmental age influences the effect of epidural dexmedetomidine on inflammatory hyperalgesia in rat pups. Anesthesiology.

[26] Premkumar LS, Ahern GP (2000). Induction of vanilloid receptor channel activity by protein kinase C. Nature.

[27] Bhave G, Zhu W, Wang H, Brasier D, Oxford GS, Gereau RW (2002). cAMP-dependent protein kinase regulates desensitization of the capsaicin receptor (VR1) by direct phosphorylation. Neuron.

[28] Parolia A, Gee LS, Porto I, Mohan M (2014). Role of cytokines, endotoxins (LPS), and lipoteichoic acid (LTA) in endodontic infection. J Dent Oral Disord Ther.

[29] Elsalhy M, Azizieh F, Raghupathy R (2013). Cytokines as diagnostic markers of pulpal inflammation. Int Endod J.

[30] Song F, Sun H, Wang Y, Yang H, Huang L, Fu D (2017). Pannexin3 inhibits TNF-α-induced inflammatory response by suppressing NF-κB signalling pathway in human dental pulp cells. J Cell Mol Med.

[31] Zanini M, Meyer E, Simon S (2017). Pulp inflammation diagnosis from clinical to inflammatory mediators: a systematic review. J Endod.

[32] Huang M-H, Wang B-S, Chiu C-S, Amagaya S, Hsieh W-T, Huang S-S (2011). Antioxidant, antinociceptive, and anti-inflammatory activities of Xanthii Fructus extract. J Ethnopharmacol.

[33] Molina C, Herrero JF (2006). The influence of the time course of inflammation and spinalization on the antinociceptive activity of the α2-adrenoceptor agonist medetomidine. Eur J Pharmacol.

[34] Vinegar R, Schreiber W, Hugo R (1969). Biphasic development of carrageenin edema in rats. J Pharmacol Exp Ther.

[35] Nazıroglu M (2015). TRPV1 channel: a potential drug target for treating epilepsy. Curr Neuropharmacol.

[36] Moran MM, McAlexander MA, Bíró T, Szallasi A (2011). Transient receptor potential channels as therapeutic targets. Nat Rev Drug Discov.

[37] Rowbotham MC, Nothaft W, Duan WR, Wang Y, Faltynek C, McGaraughty S (2011). Oral and cutaneous thermosensory profile of selective TRPV1 inhibition by ABT-102 in a randomized healthy volunteer trial. Pain.

[38] Kissin I, Szallasi A (2011). Therapeutic targeting of TRPV1 by resiniferatoxin, from preclinical studies to clinical trials. Curr Top Med Chem.

[39] Miller F, Björnsson M, Svensson O, Karlsten R (2014). Experiences with an adaptive design for a dose-finding study in patients with osteoarthritis. Contemp Clin Trials.

[40] Ma W, Quirion R (2007). Inflammatory mediators modulating the transient receptor potential vanilloid 1 receptor: therapeutic targets to treat inflammatory and neuropathic pain. Expert Opin Ther Targets.

[41] Gibbs J, Melnyk J, Basbaum A (2011). Differential TRPV1 and TRPV2 channel expression in dental pulp. J Dent Res.

[42] Chung M-K, Lee J, Duraes G, Ro J (2011). Lipopolysaccharide-induced pulpitis up-regulates TRPV1 in trigeminal ganglia. J Dent Res.

[43] Kong W-L, Peng Y-Y, Peng B-W (2017). Modulation of neuroinflammation: role and therapeutic potential of TRPV1 in the neuro-immune axis. Brain Behav Immun.

[44] Kathiria AS, Neumann WL, Rhees J, Hotchkiss E, Cheng Y, Genta RM (2012). Prohibitin attenuates colitis-associated tumorigenesis in mice by modulating p53 and STAT3 apoptotic responses. Cancer Res.

[45] Timofeeva OA, Chasovskikh S, Lonskaya I, Tarasova NI, Khavrutskii L, Tarasov SG (2012). Mechanisms of unphosphorylated STAT3 transcription factor binding to DNA. J Biol Chem.

[46] Huang FM, Chang YC, Lee SS, Yang ML, Kuan YH (2019). Expression of pro-inflammatory cytokines and mediators induced by bisphenol a via ERK-NFκB and JAK1/2-STAT3 pathways in macrophages. Environ Toxicol.

[47] Xu K, Xiao J, Zheng K, Feng X, Zhang J, Song D (2018). MiR-21/STAT3 signal is involved in odontoblast differentiation of human dental pulp stem cells mediated by TNF-α. Cell Rep.

[48] He W, Qu T, Yu Q, Wang Z, Lv H, Zhang J (2013). LPS induces IL-8 expression through TLR 4, M y D 88, NF-kappa B and MAPK pathways in human dental pulp stem cells. Int Endod J.

[49] Lee J-C, Yu M-K, Lee R, Lee Y-H, Jeon J-G, Lee M-H (2008). Terrein reduces pulpal inflammation in human dental pulp cells. J Endod.

[50] Nara K, Kawashima N, Noda S, Fujii M, Hashimoto K, Tazawa K (2019). Anti-inflammatory roles of microRNA 21 in lipopolysaccharide-stimulated human dental pulp cells. J Cell Physiol.

[51] Wang F, Han Y, Xi S, Lu Y. Catechins reduce inflammation in lipopolysaccharide-stimulated dental pulp cells by inhibiting activation of the NF-κB pathway [published online ahead of print, 2020 Jan 30]. Oral Dis. 2020. 10.1111/odi.13290. 10.1111/odi.13290 31999881

[52] Jing LU, Rong-Yin T, Yan LI. Study of primary culture method of human pulp cell. Chin J Conservative Dent. 2006;16(6):311–3.

